# Overcoming barriers to transformation in manufacturing firms. A path-dependence perspective of digital servitization

**DOI:** 10.1007/s11846-023-00641-0

**Published:** 2023-05-15

**Authors:** Thomas Brekke, Sambit Lenka, Marko Kohtamäki, Vinit Parida, Birgit Andrine Apenes Solem

**Affiliations:** 1 Jönköping International Business School, Jönköping, Sweden; 2 USN School of Business, Universitiy of South-Eastern Norway, Horten, Norway; 3 Entrepreneurship and Innovation, Luleå University of Technology, Sweden, Luleå, Sweden; 4 School of Management, University of Vaasa, Vaasa, Finland

**Keywords:** Digital transformation, Path dependence, Autonomous solutions, Digital servitization, Product–service systems (PSS), Manufacturing company, Digitalization, Organizational change, M00

## Abstract

Manufacturing firms struggle to break away from their pre-existing business models, offerings, routines, and capabilities. The present study used path dependency as a theoretical lens to investigate a single longitudinal case study of a leading manufacturing company based on in-depth interviews with senior executives and managers. The analysis contributes to extending the digital servitization and path-dependence literature by proposing four path-breaking mechanisms: (1) organizational reconfiguration, (2) reconfiguration of value offerings, (3) opportunity exploration, and (4) knowledge reconfiguration. The framework developed based on these mechanisms generated valuable insights for manufacturing firms seaking to to break away from their dominant paths.

## Introduction

Due to the pressure of de-commoditizing product manufacturing and emerging digitalization, digital servitization offers product manufacturing companies an approach to creating novel product-service-software solutions (Kraus et al. 2022; Parida Sjödin and Reim 2019). Prior studies defined digital servitization as “…the transition towards smart solutions (product-service-software systems) that enable value creation and capture through monitoring, control, optimization, and autonomous function. Digital servitization emphasizes value creation through the interplay between products, services, and software” (Kohtamäki et al. 2019, p. 383). Studies have also recognized that digital servitization promises to ensure higher performance gains and competitive advantage for product-centric manufacturing firms (Kohtamäki et al. 2022; Kraus et al. 2022). However, the extant literature on digital servitization has mainly focused on content research (Kohtamäki et al. 2019; Rabetino et al. 2018) with too little emphasis on how firms transform their routines and practices over time (Mouzas 2022; Tronvoll et al. 2020; Vendrell-Herrero et al. 2017). In this sense, scholars have called for a better analysis of how the process of digital servitization unfolds for manufacturing companies and the mechanisms responsible for resisting such transitions (Kohtamäki et al. 2021; Tronvoll et al. 2020).

Recent studies have also shown that capturing value through digital servitization is a challenging process due to the difficulty of managing the complexity of internal and external service processes, ecosystem collaboration, and the transformation of firm routines and practices (Kohtamäki et al. 2021; Suarez 2004). In fact, on some occasions, the shift toward digital servitization may lead to the failure to capture any value (Chen et al. 2021; Immelt 2017; Sjödin et al. 2020) due to manufacturers being “locked-in” to their past success and structures, which makes them resistant to adapting to contemporary development challenges (Fortwengel and Keller 2020). In this regard, there are several examples of companies that have struggled to overcome challenges posed by digital transformational, such as Hewlett Packard and its digital initiative, which lost almost five times of its investment of $160 million as a consequence of organizational inertia (Reichert et al. 2021).

Against this backdrop, the present paper examines the challenges involved in the digital transformational shift (Kraus et al. 2019) that underpins digital servitization. To investigate this sparsely researched issue, we applied a path-dependency perspective to explore how a market leader in maritime manufacturing approached this transformation. Path dependency is usually considered a characteristic that restricts what can be done, with any path changes that might occur being attributable to some shock or to purposeful agency aimed at dismantling the existing structure. Several studies have shown how superior organizations become “locked-in” by historical events and past decisions that inhibit business transformation (Garud et al. 2010; Vergne and Durand 2011). However, there is less knowledge on how such events and decisions impact the ways in which digital servitization unfolds or on how it interrupts the logic of path-dependent mechanisms by restoring access to a broader scope of action for organizational maneuverability (Kohtamäki et al. 2021; Sydow et al. 2012).

To address this gap in understanding, this paper focuses on the following research question: *How does digital servitization enable path-dependent organizations to obtain new path formations?* To answer this research question, we employed data from a single longitudinal case study of a leading maritime manufacturing company combined with data from the company’s ecosystem partners. The paper makes two main theoretical contributions. First, it identifies and discusses four path-breaking mechanisms: (1) organizational reconfiguration, (2) reconfiguration of value offerings, (3) opportunity exploration, and (4) knowledge reconfiguration, all of which are needed to ensure a successful transition to digital servitization. Second, it proposes a framework for understanding digital transformation in light of path dissolution and path formation.

The paper proceeds as follows. In the following section, we review relevant literature on digital servitization and path dependency as derived from well-known debates in business. In Sect. 3, we present the methodology we applied, including the selected research strategy, data collection technique, and data analysis process. In the Sect. 4, we present the findings of our study, after which we discuss these findings by proposing a framework for path-breaking mechanisms. Lastly, we present some conclusions and implications of our research, as well as its limitations, and additionally provide recommendations for future research.

## Theoretical background 

### Digital servitization

In the emerging digital servitization literature, increasing attention is being directed to the challenges of adapting to a digital world, such as harnessing the interplay between digitalization and servitization and incorporating digitalization into new value offerings (Kamalaldin et al 2021; Kraus et al. 2022). However, this combined concept—digital servitization—is still evolving and remains underinvestigated (Lenka et al. 2017; Tronvoll et al. 2020). Digital servitization can be considered as the utilization of digital technologies (machine learning, artificial intelligence, automation, robotics, sensors, etc.) by a company to facilitate its transition from a product-centric to a service-centric logic (Kraus et al. 2022; Raddats et al. 2019) and consequently enable value creation through the interplay between products, services, and software (Kohtamäki et al. 2021; Solem et al. 2021). The digital servitization literature highlights that a focal company’s transformation of its internal and external service processes permits value-in-use instead of a sole focus on the affordances of devices and technologies (Sklyar et al. 2019). In agreement with this line of reasoning, we believe that the transition to digital servitization requires a reorientation of company business models and internal and external processes (e.g., routines and practices) as well as better connectivity between products, services, and digital artifacts involving an ecosystem of manufacturers, suppliers, operators, and customers (Bouncken et al. 2021; Forkmann et al. 2016; Trischler and Li-Ying 2022).

According to Thomson et al. (2021), autonomous solutions constitute the most advanced representation of digital servitization in that they feature scalable systems of interconnected smart devices that function without human intervention (see also Kohtamäki et al. 2022). We assume that autonomous solutions, such as self-driven cars, buses, and unmanned vessels, transcend traditional (digital) services (in which control typically remains with humans) as they present the opportunity to make decisions and to learn and optimize processes by utilizing data from digital technologies without of human involvement. On the firm level, the introduction of autonomous solutions typically requires an organization to transform in several ways, as the focus shifts from intra-firm and customer-centric processes to a more systemic, wide-ranging arrangments that includes new forms of internal, cross-departmental collaboration, closer cooperation across industries, and the involvement of regulatory authorities, customers, and other external stakeholders as well as a shift in logic from products to value offerings (Kohtamäki et al. 2021). Consequently, this transition implies greater attention to resource and opportunity exploration, knowledge sharing, and learning through ecosystem business configuration. This in turn necessitates an increased level of system integration of solutions across a diverse and varied group of ecosystem partners, each with its own special resources (Thomson et al. 2021). In this sense, digital servitization studies view the ecosystem as an interconnected and collaborative system of business communities that co-create value through knowledge creation and the alignment of offerings around a focal firm or network of actors that respond to changes in the environment that they cannot accomplish alone (Adner 2017; Burström et al. 2021; Hou and Shi 2021; Kamalaldin et al. 2021; Kohtamäki et al. 2019; Snihur and Bocken 2022). Therefore, the strategic potential for digital servitization as a mechanism for breaking free from a locked-in path trajectory lies in identifying new sources for value creation, reconfiguring the business logic of companies and their routines and practices, establishing a data-sharing infrastructure, and changing how companies interact within an ecosystem of business actors, all with an eye toward future actions (Demil and Lecocq 2010; Palmié et al. 2022).

### Digital servitization and the path-dependence perspective

Despite the limited scholarly emphasis on digital servitization and lock-in thus far, a small, emerging stream of literature points to path dependencies as a major hurdle in organizational transformation (Bohnsack et al. 2014; Cavalcante et al. 2011).

**Fig. 1:**
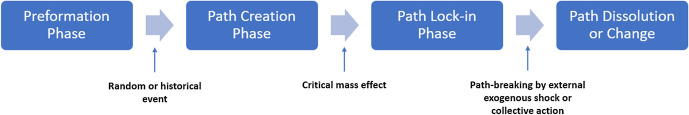
Illustration of path-dependency phases. Adapted from Sydow (2005)

According to Sydow et al. (2012), the dynamics of path dependency play an increasingly large role in economic and technological transitions, particularly in the case of complex system technologies that strive for dominance, such as autonomous solutions. In this context, we build on Sydow et al.’s (2012, p. 910) understanding of path dependency as “a sequence of economic change formed by past historical events and decision making that affect the probability of future economic development in the absence of exogenous shock or agency in the form of collective social behavior.” The trajectory of path dependence depends on past events—and is thus time-dependent—and therefore a system must follow the path it has followed up to that moment (Cavalcante et al. 2011). The shape of this path, however, is never predetermined, as exogenous random shocks, stochastic events or accidents of history, as well as potential unpredictable and nondeterminable endogenous bifurcations and systemic structural changes and transitions influence the evolution of a complex economic system (Sydow et al. 2012). Path dependency consists of three main phases (Fig. 1): (1) the preformation phase, (2) the path creation phase, and (3) the path lock-in phase. However, recent literature has also included path dissolution as a result of shocks or other events that reshape the system (Garud et al. 2010). The preformation phase is characterized by a wide range of possibilities for actions. The effect of a choice of options cannot be predicted. Once a decision is made, although it may lead to a small event, it may unintentionally initiate a self-reinforcing process with far-reaching consequences—a critical juncture. The initial choice reflects intentions (not randomly) as small events causing unintended, far-reaching consequences. The next phase is the path creation pahse. A new regime takes the lead: the dynamics of self-reinforcing processes directed by rule-guided actions and shared preferences and expectations (Arthur 1994; Garud et al. 2010; Håkansson and Waluszewski 2002). A dominant action pattern is likely to emerge, one which renders the whole process increasingly irreversible. The range of options narrows, and it becomes progressively more challenging to reverse the initial choice or the initial pattern of action—that is, a path is evolving.

Finally, the process transforms into a lock-in situation—that is, the dominant decision pattern becomes fixed and assumes a deterministic character; eventually, actions are fully bound to a path (Garud et al. 2010). One choice or action pattern becomes the predominant mode, and flexibility is lost. Primary literature from different disciplines has focused on various types of lock-in mechanisms that may be important in explaining path dependencies (Klitkou et al. 2015). Specifically, we distinguish between three sorts of lock-in mechanisms derived from the work of Arthur (1994), Grabher (1993), and Sydow et al. (2012): normative lock-in, cognitive lock-in, and resource-based lock-in.

Exogenous factors are necessary for allowing organizations to exit the narrow corridor of strategic choice, and path dissolution. Path dissolution occurs through unforeseen exogenous forces, such as shocks, catastrophes, or crises; these are likely to jolt the system, thereby causing organizations to break away from their path. According to Garud et al. (2010) and Sydow et al. (2012), transformation or de-locking might be triggered by collectivities of competent individual and/or organizational actors who coordinate their activities to expand their range of possible actions and choices. Besides the random shock argument, a path change caused by purposeful agency might take two essential forms: First, escaping path lock-in (path dissolution) might originate from actors’ initiatives within existing structures to introduce new value offerings and recombine resources, routines, and knowledge that diversify the scope of available options to challenge the existing logic of the firm. Second, path dissolution might also occur by purposeful interaction or boundary-spanning collaborations across firms, which are assumed to create a greater variety of sources and feedback so that problems and opportunities can be quickly identified and responses generated for the purpose of gaining momentum in the direction of new path formation (Kohtamäki et al. 2019).

Figure 1 illustrates the path-dependence process, consisting of three path-formation phases and a path-dissolution phase, which lay the foundation for new path formation.

## Methodology

The maritime industry is characterized by a network-centric value chain that requires collaboration across multiple business models, ecosystems, and stakeholders operating in in-/out-bound transport, ports, and sea voyages. The maritime manufacturing industry and its logistic value-chain business models operate in a dynamic and highly competitive environment and is being transformed by digital technologies, such as artificial intelligence, big data, the internet of things, cloud and edge computing, digital security, and robotics (Lorange 2009; Lorange and Fjeldstad 2010). Based on an in-depth, longitudinal case study within a large international maritime manufacturing company, we discuss the path-dependent trajectory of business model transformation in light of ecosystem evolution and digital servitization at an early stage.

### Case company description and research design

The case company is a large, multinational, Norwegian-based maritime manufacturing company operating in the B2B market. It employs 7,000 people, 36% of whom work outside Norway. The present study focused on the maritime business division, which has 4000 employees distributed among 60 local offices in 21 countries and provides platforms and equipment for 18,000 vessels. The research was conducted among senior top managers, technology and business advisors working in the different business units, and the surrounding ecosystem of business partners. The company initiative is to develop and integrate digital solutions into key operations, which involve several company subdivisions, as well as to establish new joint ventures with a global shipping operator. The research team considered this company to be suitable for the case study as it has been a global leader in the delivery of maritime automation technologies since the 1960s. Since 2016, the strategic interest of the company has been to become a global leader in the development of the most advanced form of digital servitization solutions —autonomous sea drone solutions—through joint ventures with external partners.

The research design of the present study involved an exploratory intrinsic and abductive case study approach (Creswell and Plano Clark 2007; Easton 2010), one which permitted us to detect and draw on multiple sources concerning complex firm processes sufficient for developing new insights into a novel theoretical phenomenon (Eisenhardt 1989). The research strategy built on generated knowledge from a specific contextualized digital servitization process based on a set of common theoretical constructs (e.g., path dependence and digital servitization), which established a foundation for further theory construction (Eric and Kwan 1999). The research team has been collaborating with the case company for more than 8 years. This prolonged research collaboration has facilitated excellent access to in-depth processual data, such as meeting observations and notes, interviews, strategic reports, presentations, and workshops. Employing various sources of data made the research team’s studies more accurate (Creswell et al. 2007; Denzin and Lincoln 2005) and allowed the team to develop novel perspectives and insights into the studied phenomenon of path-dependent digital servitization. Hence, the research included critical events and processes that dated back to the early 1990s, when the company was formed from several company mergers.

### Data collection and presentation

Data for the present study were collected through semi-structured interviews with company employees, former company managers, and other actors in associated ecosystems. Key informants were selected based on their involvement in technological solutions and business case development. Additional informants were identified using the snowballing technique (Coleman 1958), with key informants being asked to recommend others who might be able to offer additional insights for the study. By interviewing actors in the external ecosystem, wider insights into the phenomenon of path dependency and transformation via digital servitization were generated, as key historical events and decision making were cross-checked. The informants included former chief executive officers, departmental managing directors, business development managers, and aftersales managers, in addition to informants from customer and partner companies representing a variety of functional roles, such as managing directors and project leaders. The interviewees were asked to answer open-ended questions according to an interview guide. The interview guide was developed around overarching themes, such as narratives of important historical events and decisions concerning processes of change, technology solutions, organizational characteristics, business model architecture, and transformation (Kohtamäki et al. 2021). The interview guide was continuously revised as new insights were generated from the collected data and cross-checked with other informational sources, such as reports, webinars, and relevant literature. The research team conducted 72 face-to-face and digital interviews in total. The interviews occurred on several occasions during 2017–2022. Some of the informants were interviewed several times. Digital interviews, on Microsoft Teams and Zoom, were deployed when face-to-face interviews were not possible due to social distancing imposed during the COVID-19 pandemic. On average, the interviews, all of which were recorded and transcribed, lasted between 45 and 90 min (Table 1).

**Table 1:** Data collection method and characteristics

Data collection method	Data characteristics		
	Information	Type of data	Amount of data
Semi-structured interviews	Case company	60 interviews	60 h
	External	12 interviews	12 h
Document analysis	Reports	Company reports from 1999–2020	21 reports
	Websites	Company website information	
	Press and media	Press releases	3
	Internal presentations	Company PowerPoint presentations	10
Direct observation	Meetings	Project meetings	10
	Workshops	Company workshop	2
	Informal interactions	Short interactions (phone calls, emails, short discussions)	20
Webinars	Company	Public company webinars	6 webinars with 20 presentations

### Data analysis process

The 7 years of research collaboration and access to multiple sources generated a rich and comprehensive volume of information. The data analysis process, which was inherently abductive, examined primary (interviews, observations, transcripts, and field notes) and secondary (company reports, company presentations, press releases) data and informed the theoretical construct via an iteration process (Maxwell 2005). For independent parallel analysis and triangulation, all but one of the researchers involved in data collection also participated in coding, following guidelines formulated by Gioia et al. (2013). The one researcher not involved in coding acted as a critical contributor and thus viewed the analysis and codes with an unaffected outside-in approach. The abduction process guided the labeling of path-dependent mechanisms and lock-in categories of key stories in a way that was both theoretically and empirical grounded. When analyzing the data, the research team studied the case over time (temporal dimension), considering how historical events and decision making were impacting the business logics and transformation options of the case company. Through a series of iterations and comparisons, themes and dimensions were identified from the data. Guided by Gioia et al. (2013), a three-step process was followed. First, interviews and written information were read, and key terms were coded, resulting in first-order concepts. Second, patterns of first-order concepts led to the development of second-order themes that were on a higher level of abstraction. Last, based on the first- and second-order coding, an abstraction of aggregated dimensions was created. The NVIVO software system was used in the three-step process of coding the data into first-order categories (quotes and observations), second-order themes and patterns, and the aggregated level of dimensions (Bazeley 2007; Sorensen 2008). Throughout the study phases, the research team repeatedly compared and cross-checked the findings against relevant reports, literature, and other sources.

## Findings

**Fig. 2:**
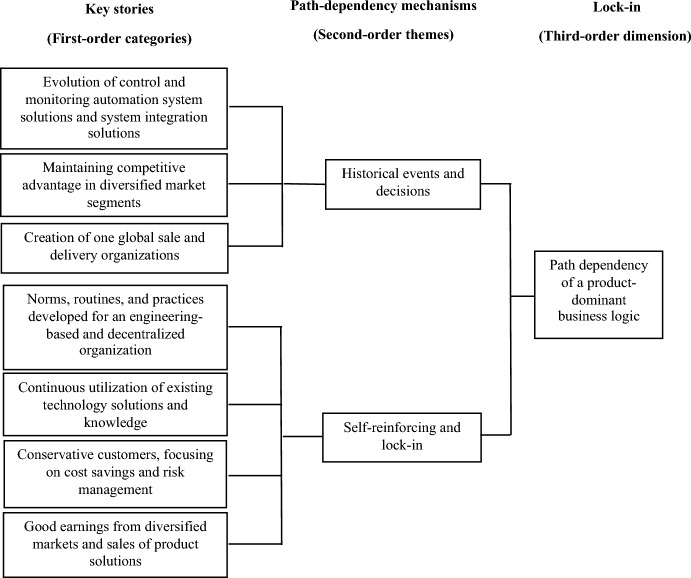
Illustration of the data structure of path-dependency mechanisms

**Fig. 3:**
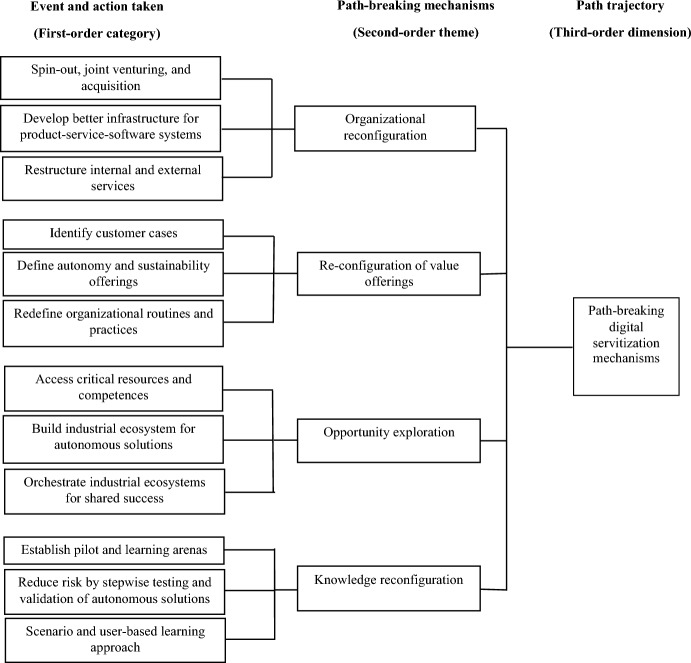
Data structure of path breaking mechanisms

This section presents the main findings of the research team’s analysis of the path dependency of the focal firm. Our findings address the progression of historical events for the case company: path formation, path creation, path lock-in, and path dissolution or change. We employed figures to visualize our findings, the generated insights, and evidence of processes of path dependency. Figure 2 is organized based on identified historical events and the lock-in of a dominant business logic. Figure 3 provides evidence of the path-breaking or de-locking mechanisms of the dominant business logic. This evidence is supported by quotes representing each of the mechanisms (lock-in and de-lock-in) at play during the digital transition of the case company.

### Path dependence, historical events, and lock-in

The empirical study concentrated on a case company operating in the maritime market. Since the 1960s and 1970s, the company has launched several ground-breaking modules of maritime automation system solutions, such as the first type of engine alarm system for domestic merchant ship fleets, a main engine control system that allows the duty officer on the ship’s bridge to control the main engine without the assistance of technicians in the engine room. In the early 1970s, the company expanded its solutions by delivering the first computerized alarm, monitoring, and control system for ship operation. These automation system solution modules gave the company a technological and market advantage, which it used to develop a fully integrated maritime control system solution. The system solution modules consisted of an architecture of systems for use in a wide range of tasks within offshore industry, oil and gas, and safety and vessel control. Based on its technology and market knowledge, the case company developed an advanced dynamic positioning system (DPS) and underwater navigation system aimed at the emerging oil and gas market in the 1980s. The DPS employs information from the company ship’s automation control systems, weather data, and wave motion data to keep the ship headed in the right direction. The DPS played an important role in the company’s global expansion and its reputation as a global technology manufacturer serving the global, ship, and offshore markets in the 1980s and 1990s. Toward the end of the 1990s, the company also included digital software solutions in its product portfolio. These solutions offered reduced operational costs by facilitating better control of onboard maintenance, spare parts/inventory control, budget control, payroll, systems, and ship-to-shore data communication systems.

The technological trajectories of the company in the 1970s to 1990s and its global expansion transformed it into a diversified firm with several strong sub-units devoted to technology and sales in the low-end and high-end markets. The global growth of the company was partly driven by structural market changes as shipyards and shipowners moved to low-cost countries, which the company followed by establishing new business units in countries such as South Korea and China. To become a global technology leader within the maritime market, the company also recognized the need to build stronger financial muscles and better coordination of its product portfolio. A strategy was launched in 1998 to increase market coordination and develop a shared company culture. To succeed with this new strategy, the company chose to reorganize its decentralized sales sub-units by creating one common global sales and delivery unit. By putting its strong sales culture in the driver’s seat, the company intended to improve its coordination of products, increase cross-departmental collaboration, and strengthen its revenue stream. As noted by one sales manager:

*It was the salespersons who were the heroes if you can put it like that. ... So, it was more sales-driven that got us further, rather than tech people having some technology and wondering where it could be used.*

Despite the success of the strategy, it also posed several challenges concerning the normative lock-in of existing routines and practices (Sydow et al. 2012). In particular, the product-oriented sales culture was dominant and shaped how development and sales were carried out. As one former product manager stated:

*The culture of selling standard systems and solutions, and because the customer knows the solution, they just want us to get the contract signed, and the culture has been like that. … If you look at our sales organization, many of them have been selling standard automation systems for 20 years, so why change when they make a lot of money from automation.*

In addition to a dominant product-oriented sales culture, the industry was characterized by conservatism, with new innovations being primarily linked to risk management and operational cost (Lorange 2009). One company R&D manager described this conservatism as follows:

*This is a conservative market too. Ship owners are not the most forward-leaning in applying new technology. … So, the ship owner will often be interested in a larger package and see the operational benefits of the operating expenses regarding maintenance.*

According to Lorange (2009), the reality of contemporary shipping is that companies must possess a portfolio of value offerings that pay substantial attention to risk management and the reduction of operational costs, and which are based on the needs and demands of clients. For a maritime equipment manufacturer, such as the case company, these issues have several implications when approaching the maritime shipping industry with novel digital solutions. First, understanding the drivers of value becomes more important than before, as only a few dominant players control the majority of the value chain. Second, identifying who bears the operational costs or how they are distributed in the value chain incorporating digitalization into new value offerings. Third, incorporating a product–service systems logic into the company’s business model will facilitate advanced control and monitoring of stand-alone system solutions with a revenue stream based on systems sales (products sales) combined with spare parts and maintenance contracts (Kraus et al. 2022; Ziajka-Poznańska and Montewka 2021).

To summarize (Fig. 2), we have identified three key historical events (development of automation system technologies, system integration solutions, and organizational change) that triggered path lock-in for the case company. We have also demonstrated how lock-in mechanisms, such as customer- and company-specific norms, routines, and practices combined with successful economic growth, were hindering the case company’s capacity to choose an alternative path for future economic growth.

### Path-breaking mechanisms via digital servitization

The findings discussed in this section progress from the data analysis of de-lock-in events and actions taken by the case company that occurred from 2016 onward. The emergent data structure and progression of the data analysis are depicted in Fig. 3. The analysis identified four de-lock-in mechanisms: *organizational reconfiguration, reconfiguration of value offerings, opportunity exploration, and knowledge reconfiguration*.

The emergence of these mechanisms and the actions taken by the company should be interpreted in light of the rapid transition of the maritime industry to a new digital operational paradigm. This digital paradigm is termed “shipping in the era of digitalization” (Ichimura et al. 2022), which is assumed to be more ground-breaking and volatile for the maritime industry than for other industries. Evidence for this argument is based on the assumption that vessel sensorization (Ichimura et al. 2022) and product-service-software systems (Solem et al. 2021) are providing a large array of data that can be used to optimize processes and operations (performance management, capacity optimizations) that are assumed to transform the broader part of the maritime logistic value chain as well as the logic of the singular firm (Munim 2019; Sanchez-Gonzalez et al. 2019). Another aspect of the digital transformation of the shipping industry is the rapid increase of connectivity by information and communication technologies (ICT), which enable new forms of business logic that open up avenues for the novel use of shipping data, thereby demanding more network-based work forms in which actors learn and co-create new knowledge and values (Ichimura et al. 2022; Tsvetkova and Hellström 2022). In addition to digitalization as a driving force, sustainability has received significantly increased attention, with the acknowledgment that new solutions must contribute to solving sustainability challenges. As one digital solutions manager stated during a webinar:

*So, talking about maritime, it is not a question of whether digitalization will impact our industry; it is commonly accepted that the adoption of digital solutions will drive us toward more efficient, safer, and greener operations. … Green shipping is for sure one of the main drivers of this technology development. You have the adoption of technology related to alternative fuels, electrification, energy efficiency, and logistics. We have only just begun to see the start of the green shipping wave, and this will drive the adoption of digital solutions in our industry.*

The shipping industry is facing the need to reduce its global footprint by lowering its dependence on fossil fuels by investing in zero-emission energy. Digitalization is seen as important for achieving the green shift, with the focus on sustainability viewed as creating new business opportunities (Parida et al. 2019). Below, we present events and actions taken by the case company that represent path-breaking mechanisms of digital servitization.

#### Organizational reconfiguration 

In 2016, the case company created a new branch devoted to digital software ventures and simulator solutions as a deliberate tactic to interrupt its inherited routines and competencies following its product-oriented logic (Chen et al. 2011). The business logic of the case company was formed over decades of successfully introducing new products for the global maritime and offshore markets driven by in-house developments combined with offerings of new product features for a lower price than its competitors. The intention was to regain its scope of options by establishing a new company entity as a step toward the development of the next generation of digital products and services (Corporate Report, 2015). The new entity was also regarded as playing a significant role in breaking free from the dominant product-oriented organizational practice, as explained by a company manager:

… *remember people are used to selling service agreements and propellers. Selling digital solutions is a whole other industry*.

The new entity was responsible for developing new digital solutions and associated technology alliances based on an already existing portfolio and revenue from advanced data, software, and simulation products. The entity was staffed with people from the engineering software departments of the case company and was supported by a group of experienced sales people. The intention was to create better resource synergies (Sydow et al. 2012) between the case company and the new entity by utilizing its global customer support network to identify potential customers and use inherited routines and competencies from the parent company. In addition to these actions, the new company became responsible for building better connectivity between analytics and product systems by permitting the large amount of ship-sensor data to be used for more advanced analytical purposes and value offerings. Enabling automation sensor data was also seen as a necessary action in terms of developing the next generation of autonomous ship solutions, such as remote operational centers (ROC) and advanced predictive software analytics. This was noted by one sales manager:

*Well, we believe it will start by connecting the vessels. There is a lot of data being generated onboard vessels today. If captured, aggregated, and used in decision making, it can help owners and operators with significant cost savings.*

The company’s effort to improve integration between software analytics and product systems was followed by the development of an ecosystem of software platforms for an emerging market of maritime applications. According to the case company, there are more than 500 maritime software applications targeting the shipping market, most of which fail to provide real business value for customers. There are several reasons for the low level of adoption. One reason highlighted by the case company is the lack of a data-sharing infrastructure between ship owners and operators that would enable faster digitalization of vessel operations. Other reasons are concerned with low data quality and functionality, which can in turn create cyber-security risks and low reliability of operational management and decision making. According to the company CEO:

*We invest a lot of money to be within the latest compliances, we are one of the first companies to develop a type of approval for remote operations and data transfer, and we are always working for a safe setup to collect and share the data or enable a customer to share the data with whom they want to share them with. … In this marketplace, we want to have a full toolkit with a variety of applications that cover all the needs a shipper has.*

The above quote emphasizes the company’s ambition to become the main digital connectivity provider for the maritime industry by offering a toolkit for utilizing data for software analytics based on company-specific competencies and routines for approved data-sharing methods. The quotation also highlights the company’s efforts to regain its scope of actions by organizational reconfiguration (e.g., new business units) and the transition from a product–service-logic to a product–service-software systems logic.

#### Reconfiguration of value offerings

The second identified path-breaking mechanism concerns the capability of the company to invent and introduce new value offerings for the maritime market within autonomous shipping. According to Munim (2019), major ship actors look for innovative solutions that can increase the demand for shipping by penetrating new markets through a modal shift of cargo from road to sea. Autonomous ships are regarded as playing such a significant role as they allow ships to independently control their own actions while transporting goods from one port to another and also reduce operational costs (Ziajka-Poznańska & Montewka 2021).

Findings from the analysis show that several events and actions taken by the company increased its maneuverability toward creating a new path trajectory based on autonomous ship solution offerings. Informants highlighted several research reports and analyses in the mid-2010s from which they drew lessons concerning the introduction of autonomous vehicles in the car market and the extent to which introducing such autonomy to the maritime industry could be significant. Based on these reports, a small group of engineers within the case company began exploring autonomy in the context of shipping by building some pilot tests and conducting a concept analysis. The group of engineers discovered that the company’s existing domain of knowledge and technologies formed an excellent foundation for integrating existing system solutions with new autonomous features. However, the group received little support from the company’s middle management, as autonomous solutions were regarded as an immature technology with no real customer cases and were many years away from being commercialized. However, despite reluctance on the part of middle management, news about the pilot tests caught the attention of a national retailer and logistics company, which asked the case company for help in building and operating an autonomous short shore carrier vessel. The retailer’s initiative was based on its zero-emission strategic ambition to reduce its carbon footprint by investing in new sustainable and zero-emission solutions. The request for assistance from such a large logistic retailer increased attention to and support from the company’s top management and justified the significantly larger investment in autonomy and digitalization by the case company. This, in turn, laid the groundwork for actions taken by the case company to assume a leading developmental role as a system integrator of autonomous solutions. As one business manager explained:

*… we saw that we had a lot around dynamic position and navigation, and we had the communication systems as well. We saw that we maybe were the company that was best suited to go into this area.*

Our analysis demonstrated how forward-looking customers play a significant role in promoting the implementation of new technology.

Gradually, a new path opportunity emerged as the case company began collaborating with a new and progressive customer—not a conservative maritime customer, but one focused mostly on cost reduction and risk management as the key driver of change. Identifying such early adopters became more critical for the case company, as doing so might confirm the reliability of the technology and the existence of a market as well as serve as a means to explore unique value offerings for digital customers. According to Munim (2019), autonomous ship solutions consist of next-generation modular control systems and communications technology that enable wireless monitoring and control functions both on and off board. They also include advanced decision support systems that provides the capability to operate ships remotely under semi or fully autonomous control. It seems apparent that digitalization in terms of building autonomous solutions is a complex and demanding process, one that the following business manager characterized as a challenge related to the development of their current organizational routines and practices:

*… just in the last year, we have made dramatic changes in the organization, autonomy is just one mechanism. Autonomy requires us to be leaner, better integrated, and adapted to be able to meet the demands of the future. Big change is happening internally; autonomy is a catalyst for that.*

The above quote underscores that autonomy is causing organizational change in the company by reconfiguring organizational routines and practices and fostering new forms of cross-departmental learning and knowledge-sharing activities aligned with unique customer needs (Kohtamäki et al. 2019; Sjödin et al. 2020). Skills required to adapt to changing demands and integrate new knowledge or insights into the broader organization seem to be essential for the case company to maintain its competitive advantage regarding the reconfiguration of value offerings (Solem et al. 2021).

#### Opportunity exploration

In addition to these above-described path-breaking mechanisms, the analysis revealed how the case company explored new opportunities by forming an industrial ecosystem aimed at developing, testing, and implementing advanced digital product–service solutions (Adner 2017). According to Kohtamäki et al. (2019), digital servitization calls for better collaborations (accessing resources) and the integration of value offerings across the boundaries of firms to more comprehensively explore new market opportunities, which a singular firm cannot do alone. Another aspect concerns the regulatory acceptance of a different level of autonomy (from manned to fully autonomous) and competencies within ship compliance, such as monitoring and reporting national and international requirements. The development and introduction of autonomous systems proved to be a very demanding and complex process when it came to laws, national and international regulations, security, compliance, and operational risk management. As a maritime technology manufacturer, the case company did not possess the requisite knowledge nor did it have sufficient approval (compliance). Therefore, it launched a joint venture with a ship operator and compliance company. As one manager argued:

*… the joint venture company was established to handle autonomous ships to operate them. It is a joint venture between the ship operator and us because we had to bring in some knowledge from shipping. They were supposed to feed the back-office services through the shore control center, the body holding the certificates, the complement rules, and regulations. They would also feed the people working in the shore control center with knowledge. They have experienced ship officers and a logistic network throughout the world. So that was also something to take advantage of through that cooperation.*

Another action taken was the orchestration of an industrial ecosystem of actors*.* As the speed of digitalization of maritime shipping logistics was increasing and more competitors were entering the market, the case company decided, after a few years, to open its digital infrastructure and platforms to other users by signing a strategic partnership with several prominent maritime players. Its purpose in doing so was to increase the network effect of its digital infrastructure platforms by increasing data access to create more business opportunities.

Still, the digital transformation and, in particular, ship autonomy remains in a very early phase of development. It has yet to be scaled into commercial activity, and most related initiatives are still in the pilot testing phase (see Munim 2019), which is aimed at addressing who is in the best position to build and scale autonomous solutions. According to the case company, there are only a few actors who have the capacities, knowledge, and skills needed to build, test, and implement such complex and advanced systems. The case company saw itself as well positioned to assume the leading role as an ecosystem orchestrator for autonomous solutions*.* This issue was highlighted by a sales director:

*… so, we are one of those who are trying to take that position. Both in the form of system integration, but also in relation to digitalization … data collection, data sharing, data analysis, that we also have a grip on the infrastructure. But we see that we will be wasting much time if we try to do everything ourselves without collaborating, and that means we need to go into open source, providing an open and common platform.*

This statement shows how successfully introducing digital solutions depends on the willingness of actors to share resources and their experience of getting something in return (Kamalaldin et al. 2021; Leminen et al. 2022), which demonstrates the delicate role played by the company in balancing and ensuring joint success.

#### Knowledge reconfiguration

As mentioned in previous analyses, the maritime market primarily consists of conservative customers who mostly focus on cost reduction and risk management as the main value offerings. The case company quickly realized that selling advanced digital service solutions was different from selling products and maintenance contracts in a conservative maritime market. First, the main differences concern the high complexity of other systems collecting data from the vessel and building a new ROC that can operate and maneuver ships. Second, there were several different actors, such as technology suppliers, shipbuilders, ship owners, harbor owners, and operators, and different types of authorities who had to work together to solve many of the challenges that accompany new innovative autonomous solutions. Overall, the complexity of the technology and the coordination of industrial actors and regulatory authorities necessitated the application of an experimental learning approach, one in which customer needs were foregrounded in the learning process as a source of reflection and adjustment of proposed solutions. As one sales manager stated:

*… because no one has done that much digitalization yet, they understand they need to do something, but they do not understand what to do.*

Another challenge experienced by the partners was that the regulations and those who enforced them needed to be sufficiently prepared and mature to be able to take the full step from a manned ship to a fully unmanned ship. Regarding this issue, the sales director stated the following:

*There is no doubt, this is a maturity process both for the regulations, technology, sales, and deliveries. Vessels without people is something else than vessels with people.*

Building acceptance from authorities and regulatory bodies became a critical juncture for the project. A stepwise trial-and-error process was applied to gain experience, to learn, and to make necessary adjustments. The purpose was to test and validate the solutions on each level of autonomy (fully manned, crew on board, and unmanned) before being put into commercial use. The step-by-step approach allowed authorities to grant limited dispensations from the legislation so that testing could be carried out in a real environment. This process was reflected upon by a product manager:

*It is a long period of testing with real-life scenarios with a crew onboard and gradually allowing the automated systems to take over more and more of the operation, until the legislative bodies feel satisfied that it is a safe operation, and they will grant approval for the vessel.*

The step-by-step approach was also important for enabling the customers and actors to become engaged and co-create value, as well as to learn and correct errors from each of the development phases, as one sales director emphasized:

*It is a bottom-up process where you get together with the customer and enable them to create added value to get a sort of return on the investment. … This is a step-by-step process. We build the different functions and operations based on experience, and of course we are making a lot of mistakes, but we try to learn from them.*

The case company, the partners, and the customers co-created a digital service innovation (Sjödin et al. 2020; Solem et al. 2021) by applying an experimental development process based on the step-by-step piloting and testing of solutions in close collaboration with customers and stakeholders. Such an experimental development process requires the actors to adapt their solutions in line with those of others for the purpose of both anticipating and complying with future laws and regulations so that the solution can meet the requirements of the customer. Such a co-creative work arrangement affords participants a richer base of experience in that they are able to discuss and reflect on different perspectives concerning the best possible solutions (Westling et al. 2019). Furthermore, the quotations of this section emphasize how the company is reconfiguring its knowledge and learning processes by adapting and adding customer and stakeholder experiences into an co-creative work arrangement. Knowledge reconfiguration does not necessary modify the company’s underlying structure, such as technology platforms, customer segments, or business functions. Instead, knowledge reconfiguration increases the company’s capability to disrupt its “sticky” routines and practices that undermine growth—or to change strategic direction in the face of major industry transformation.

Figure 3 below summarizes our findings on how path-breaking mechanisms helped to de-lock the dominant business logic of the case company by cultivating a path-deviant behavior, one that permitted digital servitization linked to four identified path-dependency capabilities: (4.2.1) organizational reconfiguration, (4.2.2) reconfiguration of value offerings, (4.2.3) opportunity exploration, and (4.2.4) knowledge reconfiguration.

## Discussion. A framework for path-breaking mechanisms

**Fig. 4:**
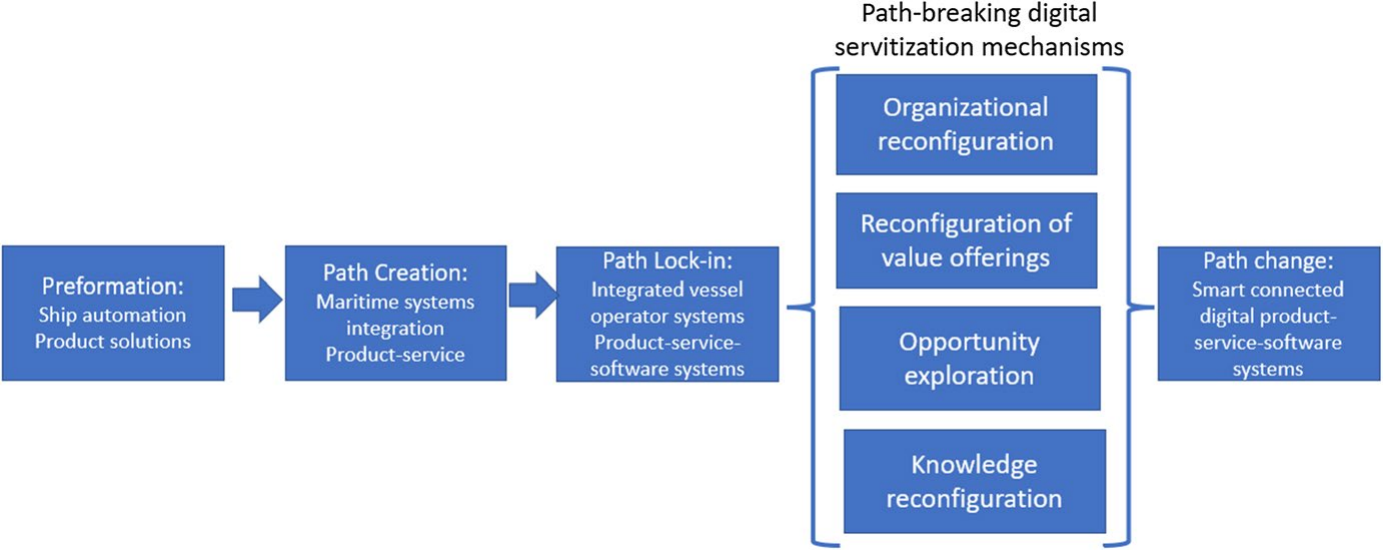
Path-breaking digital servitization mechanisms

Figure 4 illustrates the path-breaking mechanisms generated from the analysis of the literature and activities reported by the focal case company. The framework visualizes the maritime manufacturer’s four path-breaking mechanisms: digital connectivity and infrastructure, autonomous solution capacities, industrial ecosystem, and experimental learning. Based on Fig. 4, Table 2 shows how these path-breaking mechanisms affect path dissolution and formation. We contend that the framework and the table can serve as a guiding principle for increased path maneuverability or for the dissolution of the existing path by broadening the variety of business opportunities afforded by digital servitization. In the following sections, we explain in detail the logic underlying each path-breaking mechanism and its effects.

**Table 2:** Path-breaking mechanisms

Cause	Mechanisms	Effect	Indicator
Connectivity	Organizational reconfiguration	Restructuring	Spin-out, joint venturing and acquisitions, consortia
		Digital infrastructure	Software analytics, digital platform(s), Product-service-software systems
		Customer insights	Extent of customer involvement
Autonomy	Reconfiguration of value offerings	Cost savings	Reduce operational costs and human error
		Environmental upgrades	Zero emissions, decarbonization
Ecosystem	Opportunity exploration	Exploit new opportunities	Role alignment
		Access new capabilities	Resource exploitation
		Shared success	Procurements, contracts, revenue models
Co-creation	Knowledge reconfiguration	Piloting and testing	Stepwise validation, stakeholder involvement
		Experimental learning	Competence upgrades, widening of the experience base, change of routines and practices

### Organizational reconfiguration—escaping from the past

While the first phase of the digitalization of shipping consisted of gradually turning ship automation product solutions into integrated vessel operator systems, the next phase represented a much more advanced level of digitalization of the shipping industry based on better connectivity, which is seen as a significant game changer for the shipping industry (Fig. 4) (Kohtamäki et al. 2021; Thomson et al. 2021). Consequently, the maritime manufacturer is facing several challenges concerning its past investments and decision making regarding superior technologies, organizational efficiency, and the accumulation of a large customer base, which have created a successful, but locked-in, path evolution. To regain the scope of options and stimulate better maneuverability, the case company spun-out its most advanced digital services into a new unit, performing joint venturing, active acquisitions, and the building of a corporate digital infrastructure. This action was assumed to strengthen the potential for creating a new alternative path for the company. Here, organizational reconfiguration serves as a break-up mechanism, separating the company from its past events and actions and its inherent routines and practices. However, links to the parent company were maintained, such as its customer base and delivery organization, which led to the development of new routines and practices, including new sales practices.

### Reconfiguration of value offerings—expanding possibilities for actions

Building advanced digital shipping services—the autonomy trend—involved the configuration of new sources of value offerings based on cost savings (reduced manning and operational costs) and environmental upgrades (zero emissions) (Parida et al. 2019). As the analysis identified, introducing autonomous solutions in a conservative shipping market was made possible by the fact that an external industry player, a logistics retailer, saw new business opportunities that traditional shipping players were unable to perceive. First, the curiosity and initiative of a first mover (such as the retailer and logistics company) made it possible for the case company to move from the ideation phase to piloting and implementation. As the first mover was not familiar with the shipping branch and the solutions were new to the industry, the retailer needed more advisory support based on an experimental and trust-based form of work. Second, autonomy as a value proposition first gains momentum when primarily sustainability goals are linked to value propositions, such as better utilization of resources and the reduction of emissions. As the analysis showed, combining autonomy and sustainability value offerings with new types of customers compelled the case company to rethink its value offerings concerning autonomy and to assume a new and more prominent role as a systems integrator for the delivery of advanced digital service solutions.

### Opportunity exploration—diversifying scope of options

As firms become more involved with advanced digital service offerings through ecosystem involvement (Kamalaldin et al. 2021; Kohtamäki et al. 2019), they also become more dependent on the resources and actions of other actors. Aligning and orchestrating the actions, resources, and revenue model of partners helped to ensure a win–win scenario: shared success and mutual access to unique resources that broadened the scope of action of each individual company. For some of the ecosystem actors, their motives and ambition to participate were based on the assumption that the project would give them a competitive advantage and new business opportunities. The continuous search for sources and the incorporation of external resources through ecosystem collaboration were found to be a demanding process, as existing organizational cultures and practices must be aligned and adjusted to utilize shared resources and shared knowledge (Tsvetkova and Hellström 2022). In this sense, opportunity exploration acts as a mechanism for actors’ search for new opportunities, re-use of shared resources, and transfer of competencies as the basis for new growth.

### Knowledge reconfiguration—direction of new path formation

Advanced digital services in the maritime and shipping market depend heavily on the co-creation of learning and knowledge (Kohtamäki et al. 2021; Lenka et al. 2017; Sjödin et al. 2020), which increases the diversity of technological and economic knowledge and hence the potential for introducing new solutions in a conservative and well-regulated shipping market. Apart from the fact that the shipping market is characterized by conservatism, the shipping industry is also heavily regulated by national and international laws and regulations intended to guarantee the safety of crew and personnel. Accordingly, all new solutions must comply with these laws and regulations before they can be implemented. Therefore, introducing autonomy would entail significant changes and adaptations to existing laws and regulations before a higher level of autonomy (from manned to unmanned vessels) can become a reality. To do so—and to convince the conservative shipping market—the case company employed an experimental and exploratory development process that required the step-by-step trial and testing of solutions designed to increase the competence and experience of the actors. Another finding demonstrated how an internal, progressive group of employers can drive a company transition by searching for and recombining existing knowledge (automation and ship integration) and routines into the future domain of opportunity (advanced digital services). Learning by a stepwise experimental approach acted as a feedback mechanism by which actors had to reflect on challenges and adapt to other solutions by co-creating new knowledge (Tronvoll et al. 2020). New skills in governing such complex, uncertain, and high-risk projects based on co-creation requires good reflexive capacities that permit one to account for the solutions proposed by others with the intention of determining and promoting the best solutions for the collective good of the community, in turn laying the foundation for new path formation.

Table 2 summarizes some of the identified key forces that cause changes (connection, autonomy, ecosystem) in the contemporary shipping industry. Table 2 also outlines four de-lock-in mechanisms and their path-dependent enabling effects, as well as indicators of actions that companies can take to change a locked-in position.

## Conclusions and implications

The present study set out to discuss the path dependencies, path-lock-in mechanisms, and path-breaking mechanisms of manufacturing firms. We employed an in-depth single case study to analyze a product-service-software company’s path dependencies and path-breaking mechanisms to answer the following research question: *How does digital servitization enable path-dependent organizations to obtain new path formation?* Our findings highlight several aspects of digital servitization-based transformation and path-breaking mechanisms, and thereby contribute to several streams of literature.

### Theoretical contributions

First, we contribute to the digital servitization literature by applying the path-dependency lens to explain some of the challenges faced in implementing a digital servitization strategy in product manufacturing companies (Kamalaldin et al. 2021; Kohtamäki et al. 2022; Thomson et al. 2021). Second, our study explicated four path-breaking mechanisms of a manufacturing company: (1) organizational reconfiguration, (2) reconfiguration of 0 value offerings, (3) opportunity exploration, and (4) knowledge reconfiguration. These mechanisms are deployed to break free from the past focus solely on product manufacturing. The transition is far from easy, and manufacturers are advised to find their own effective ways to initiate such critical path-breaking mechanisms. By identifying path-breaking mechanisms in the context of digital servitization, we provided a micro-foundation-based explanation (Vergne and Durand 2011) for successful implementation of a digital servitization strategy, which is not very well understood and analyzed either in the digitalization servitization (Kothamäki et al. 2019; Rabetino et al. 2018) or path-dependence literature (Sydow et al. 2012). Third, our study revealed mechanisms that reinforce path dependency, such as historical events related to the creation of the product manufacturing business logic and the self-reinforcing mechanisms that generate lock-in (Arthur 1994; Grabher 1993; Sydow et al. 2012). Lastly, we contribute to the path dependency literature by identifying underlying mechanisms responsible for triggering a new path development, which explains in detail some of the evolving dialogue of path change as deliberate collective action (Garud et al. 2010; Sydow et al. 2012) or an external shock of some kind (Arthur 1994; David 2005).

### Managerial implications

Overall, product manufacturing is an industry that has faced significant digitalization during the past 2 decades. The transition is still ongoing—and accelerating. New digital technologies are providing expanded and novel opportunities for manufacturers to explore and exploit. The shift toward digital servitization has been challenging, with many companies continually struggling to change their strategies, business models, organizational structures, and culture. This study helped provide an understanding of the events and actions that culminate in path dependency, from which it is difficult for manufacturing companies to break free. The study also outlined a clear framework for how manufacturing companies can adopt the identified path-breaking mechanisms to help implement their own digital servitization strategy.

The findings of this study yielded insights that may be of great value to practitioners and managers in seeking to understand the mechanisms of both path lock-in and path de-lock-in—not only on the level of individual firms but also at the interfirm and branch levels as well. Such enhanced understanding may also be crucial in terms of solving major sustainability-related challenges, some of which require faster digital transformation, such as transitioning from fossil fuels to zero-emission sources. Policymakers may also find the findings and contributions of this research useful, especially in conceptualizing how digital servitization might be employed to achieve climate goals and also which historical events could affect the capacity of the company to adapt and become more sustainable.

### Limitations and suggestions for future research

The present study was based on a case study of digital servitization within a single maritime manufacturing company. Although the empirical basis for our findings is fairly broad, we acknowledge that future research on autonomous solutions may be contingent on, for instance, industry differences. Cultural disparities and differing regulations between companies, company branches, and countries may all play a role in the design of business models aimed at delivering autonomous solutions. Consequently, we recommend that further research should account for such variations. In addition, we hope that future research will validate the path-dependence framework we have developed to lend support to our findings. Studying the mechanisms of path dependence (lock-in and path breaking) with respect to the development of autonomous solutions and the associated business model concept in other contexts could provide additional or novel insights that allow the generation of a more generalizable framework. Likewise, regulatory issues concerning the acceptance and implementation of autonomous solutions should be further investigated to better understand the transformational challenges involved in such processes.

Furthermore, our study laid the foundation for future research on how to develop a path dependence-based understanding of digital servitization as well as why some companies may fail to develop a new path direction, while others succeed. Capturing the path phases and activities that equipment manufacturers undertake when more advanced digital transitions are to be achieved, such as complex systems of autonomous solutions, will equip managers with more decisive knowledge and valuable insights into path-breaking mechanisms. Similarly, the actions required to reconfigure and transform a dominant business logic within an ecosystem of business communities can be further investigated in future studies. Doing so would open up new and interesting research avenues with regard to the ways in which actors can collaborate and co-create value offerings, and how constellations of ecosystems can capture value by balancing the roles of different ecosystem actors.
